# BMI1, a new target of CK2α

**DOI:** 10.1186/s12943-017-0617-8

**Published:** 2017-03-07

**Authors:** Soumyajit Banerjee Mustafi, Prabir Kumar Chakraborty, Shailendra Kumar Dhar Dwivedi, Kai Ding, Katherine M. Moxley, Priyabrata Mukherjee, Resham Bhattacharya

**Affiliations:** 1 Peggy and Charles Stephenson Cancer Center (OUHSC), University of Oklahoma Health Science Center, 975 NE 10th Street, BRC-1409B, Oklahoma City, OK 73104 USA; 2 Department of Obstetrics and Gynecology, University of Oklahoma Health Science Center, Oklahoma City, OK USA; 3 Department of Pathology, University of Oklahoma Health Science Center, Oklahoma City, OK USA; 4 Department of Cell Biology, University of Oklahoma College of Medicine, Oklahoma City, OK USA; 5 Department of Biostatistics and Epidemiology, University of Oklahoma Health Sciences Center, Oklahoma City, OK USA

**Keywords:** Post translational modification, Phosphorylation, Kinase assay, Protein stability, Formalin fixed paraffin embedded tissues (FFPE), Fallopian tube epithelial (FTE) cells, High-grade serous ovarian cancer, Clonal growth

## Abstract

**Background:**

The polycomb group protein, BMI1 plays important roles in chromatin modification, stem cell function, DNA damage repair and mitochondrial bioenergetics. Such diverse cellular functions of BMI1 could be, in part, due to post-translational modifications, especially phosphorylation. To date, AKT has been reported as a kinase that by site specific phosphorylation of BMI1 modulates its oncogenic functions.

**Methods:**

Immunoprecipitation in conjunction with kinase assay and mass spectrometry was used to determine association with and site specific phosphorylation of BMI1 by CK2α. Functional implications of the BMI1/CK2α axis was examined in cancer cells utilizing siRNA and exogenous gene expression followed by biochemical and phenotypic studies. Correlations between expression of CK2α and BMI1 were determined from cell lines and formalin fixed paraffin embedded tissues representing the normal fallopian tube epithelium and high grade serous ovarian cancer samples.

**Results:**

Here we report that CK2α, a nuclear serine threonine kinase, phosphorylates BMI1 at Serine 110 as determined by in-vitro/ex-vivo kinase assay and mass spectrometry. In ovarian cancer cell lines, expression of CK2α correlated with the phospho-species, as well as basal BMI1 levels. Preventing phosphorylation of BMI1 at Serine 110 significantly decreased half-life and stability of the protein. Additionally, re-expression of the phosphorylatable but not non-phosphorylatable BMI1 rescued clonal growth in endogenous BMI1 silenced cancer cells leading us to speculate that CK2α-mediated phosphorylation stabilizes BMI1 and promotes its oncogenic function. Clinically, compared to normal fallopian tube epithelial tissues, the expression of both BMI1 and CK2α were significantly higher in tumor tissues obtained from high-grade serous ovarian cancer patients. Among tumor samples, the expression of BMI1 and CK2α positively correlated (Spearman coefficient = 0.62, *P* = 0.0021) with each other.

**Conclusion:**

Taken together, our findings establish an important regulatory role of CK2α on BMI1 phosphorylation and stability and implicate the CK2α/BMI1 axis in ovarian cancer.

**Electronic supplementary material:**

The online version of this article (doi:10.1186/s12943-017-0617-8) contains supplementary material, which is available to authorized users.

## Background

BMI1 is a member of the polycomb repressor complex 1 (PRC1) that in complex with Ring1B catalyzes mono-ubiquitination of histone H2A (H2AK119) and mediates gene silencing by regulating chromatin structure. Accumulating evidences have established important roles for BMI1 as a transcriptional repressor, a regulator of stem cell function [1, 2], a DNA damage repair protein [3–5] and recently, a mitochondrial role has been described [6, 7]. The importance of BMI1 is further underscored by the fact that it has been implicated in several different malignancies including ovarian cancer leading to the development of clinical small molecule inhibitors [8–10].

The BMI1 protein comprises of 326 amino acids with an N-terminal RING finger (RF) domain, a middle helix turn helix (HTH) domain and a C-terminal Proline-Serine rich (PS/PEST) domain [11]. Post-translational modifications confer functional diversity to proteins and few such modifications have been described for BMI1 [3, 5, 12–14]. When phosphorylated by 3 pK (MAPKAP kinase 3), specific sites unknown, BMI1 dissociated from the chromatin resulting in de-repression of targets [13]. Phosphorylation by AKT at Ser 316 of BMI1, triggered its dissociation from the INK4A/ARF locus resulting in inability to promote tumor growth [12]. On the other hand, AKT-mediated phosphorylation of BMI1 at Ser 251, 253 and 255 enhanced its oncogenic potential in prostate cancer [3]. It is notable that the described phosphorylation sites reside within the PS/PEST domain, deletion of which enhances stability of the BMI1 protein [15]. The functional fate of phosphorylated BMI1 thus remains context and cancer specific.

Like BMI1, the Ser/Thr kinase complex, Casein Kinase 2 (CK2), is involved in various cellular processes affecting clonal growth, survival, apoptosis and DNA damage repair [16–18]. Similar to BMI1, CK2 is predominantly present in the nucleus and comprises of a hetero-tetramer of the catalytic α, α’ and two non-catalytic β subunits [18]. Notably, expression of both CK2α and BMI1 is elevated in ovarian cancer and correlates with poor overall survival [19–21]. Together with predictive bioinformatics analysis, these evidences prompted us to evaluate CK2α as a potential kinase in phosphorylating BMI1. Here, we establish that CK2α is a novel kinase for BMI1; CK2α phosphorylates BMI1 at Ser110, and this phosphorylation imparts protein stability contributing to clonal growth. The identified CK2α-BMI1 liaison is of clinical significance as high CK2α expression strongly correlates with the elevated BMI1 protein levels in high grade serous ovarian cancer tissues. Our results implicate the CK2α/BMI1 axis in ovarian cancer, thus targeting it might be potentially beneficial.

## Methods

### Materials

Lambda phosphatase was purchased from New England Bio labs (#P0753S). Casein Kinase II Inhibitor I, TBB was purchased from Calbiochem (#218708). CK2α-GST tagged or CK2α untagged recombinant proteins were purchased from Signalchem (#C71-10G-10) and Abcam (#90745) respectively. GST-tagged or full-length BMI1 recombinant proteins were purchased from Mybiosource.com (#MBS717171) and Origene (TP760041) respectively. GST was purchased from Abcam (#89499). Nonradioactive ATP (Adenosine triphosphate) was purchased from Thermofischer (# R1441) and radioactive ATP labeled on the gamma phosphate group with ^32^P was purchased from PerkinElmer (#NEG002A250UC). Cyclohexamide, MG132 and AKT inhibitor (Akti) were procured from Sigma (#R750107) and Calbiochem (#474791; 124018) respectively.

### Plasmids and constructs

The plasmids FLAG-BMI1WT (wild-type) construct as previously described (Fan et. al. 2008), was used to generate S110A mutant BMI1 FLAG. Quick Change Site directed mutagenesis kit (Agilent) and the following pair of primers were used to generate the mutant. Both the plasmids were sequenced and the integrity was confirmed before performing experiments. (Forward primer: CTGATGCTGCCAATGGCGCTAATGAAG ATAGAGGA; Reverse Primer: TCCTCTATCTTCATTAGCGCCATTGGCAGCATCAG). pZW6 (CK2α) was a gift from David Litchfield (Addgene plasmid # 27086) [22].

### Cell culture and transfection

CP20 cells (developed by sequential exposure of the A2780 parental cell line to increasing concentrations of cisplatin) and OV90 cells were kindly gifted by Dr. Anil Sood, MD Anderson Cancer Center. OVCAR4 was a kind gift from Dr. Ronny Drapkin, formerly at the Dana-Farber Cancer Institute, Boston, MA. Immortalized normal fallopian tube epithelial cells (FTE-188) were a kind gift from Dr. Jinsong Liu, MD Anderson Cancer Center. OVSAHO and TKYNU cell lines were obtained from the Japanese Collection of Research Bioresources Cell Bank (JCRB). These were routinely cultured in RPMI/DMEM/MCDB-Med199 (Sigma) supplemented with 10% FBS and 1X penicillin-streptomycin (Gibco, NY, USA) in a 5% CO_2_ humidified atmosphere.

Lipofectamine 3000 (Invitrogen) was used for plasmid transfection and co-transfection with siRNA as per manufacturer’s protocol. Gene silencing was performed using Hiperfect (Qiagen) and 33 picomoles siRNA (scrambled control, Dharmacon; *BMI1* SASI_HS01_00175765 from Sigma and CK2α siRNA #6389, CST). All experiments were performed 48–60 h after transfection, unless stated otherwise.

### Protein extraction, determination of protein concentration, and λ-Phosphatase treatment

Total Cell Lysate was prepared in RIPA (Boston Bioproducts) or Cell Lytic M (Sigma) compatible with enzymatic assay and immunoprecipitation.

For the formalin fixed paraffin embedded (FFPE) patient sample, samples were prepared as previously described with slight modification [23]. First, samples were deparffinized with 1 ml of xylene; vortexed and stored for 10 min, followed by centrifugation at 14,000 g × 5 min and then supernatant was removed. Deparffinization process was repeated thrice following which samples were gradiently hydrated starting from 100% ethanol followed with 80% and 50% ethanol. After each step the samples were centrifuged at 14,000 g × 5 min and supernatant was removed. Samples were stored overnight at 4 °C in 1 ml of DEPC-treated water and then centrifuged at 14,000 g × 15 min and the pellet was resuspended in 2% SDS buffer with brief pulse of sonication (10 s × 3 times), centrifuged (14,000 g × 15 min) and supernatant collected for downstream assay.

Concentration of the extracted protein was determined by Bichonic acid assay (BCA) method using Pierce Kit (#23225). For most assays 10–30 μg of the lysate was used.

λ- Phosphatase treatment was performed on 50 μg of cellular protein incubated with 200units of the phosphatase at 25 °C for 1 h as per the manufacturer’s protocol. Reaction was terminated by addition of the 4 × lamelli buffer.

### Immunoblotting, immunoprecipitation and kinase reaction

Immunoprecipitation was performed using Agarose A/G beads (SCBT-2003) or the Pierce crosslink IP kit (Cat No # 26147) and cell lysates or immunoprecipitated proteins were separated by SDS-PAGE and Western immunoblotting analysis was performed using standard protocol as described previously [24]. The cell lysate were separated on 10 or 12% glycine SDS-PAGE gel or Phos-tag™SDS-PAGE (for analysis of BMI1 phosphorylation in a panel of normal and high grade serous ovarian cancer cells; Wako Cat No # 304-93521). For Mn^2+^-Phos-tag SDS-PAGE, 50 μM Phos-tag™ acrylamide and 2 eq of MnCl_2,_ when added to the resolving part of 10% acrylamide gel provided a convenient method for the simultaneous analysis of a phosphoprotein isoform(s) and its non-phosphorylated counterpart [25–27]. Gels were transferred to PVDF membrane. Membranes were blocked in 5% non-fat dry milk in TBS with 0.1% TWEEN-20 (TBST) for 1 h at room temperature followed by incubation with indicated primary antibodies in TBST with 5% BSA. Antibodies were purchased from following venders: Santa Cruz Biotechnology (CK2α #sc-373894, Akt1/2/3 #sc-8312, p-Akt1/2/3 (Ser473) #sc-7985); Proteintech (HSP60#66041-1-Ig); Life-Technologies (BMI1#375400); ABCAM (α − Tubulin #ab4074) and Sigma (FLAG #F1804, secondary antibodies conjugated with horseradish peroxidase IgG Rabbit and Mouse).

10 μg of required antibody (BMI1 or CK2a) was first crosslinked to resin using DSS following crosslinking protocol of the manufacturer (Pierce, crosslinking IP protocol). Alternately, 1 mg of precleared lysate was incubated with the antibody overnight at 4 °C in spin wheel. The beads were added to the protein/antibody homogenate on the next day and incubated at 4 °C for 1 h in a spin wheel. The beads were collected by centrifugation, washed and suspended in 15 μL IP buffer to be used for radioactive or nonradioactive kinase assay. Radioactive assay for CK2α was performed using 10 μg of BMI1/BMI1-GST with 9 μl of final immunoprecipitate bead in 15 μl reaction mix respectively containing 50 mM Tris–Cl pH 8.0, 10 mM MgCl2, 50 μM ATP and 5 μCi of [γ-32P] ATP (3000 Ci/mmol). The reactions were carried out for 15 min at 25 °C after which they were stopped by adding 4 × samples loading buffer. The samples were subjected to SDS-PAGE, the gels were dried and exposed at −80 °C as required on an X-ray film and developed. Non-radioactive assay for CK2α used similar protocol with only the radioactive ATP being replaced by 200 mM of cold ATP and BMI1/BMI1-GST being used as substrate. The films were scanned and quantified using Image J software.

### Quantitative RT-PCR

Cellular RNA was isolated (Zymo RNA isolation kit, Cat No: R1050) and 1 μg of total RNA was reverse transcribed to cDNA (iScript cDNA synthesis kit, Bio-Rad). Total of 1 μl of the 40 μl reverse transcription reaction were used as template for real-time detection (Bio-Rad CFX connect) using Sybr green (Bio-Rad) and primers as described below. Gene expression was quantified for the tested genes and endogenous control gene 18 S rRNA. Relative mRNA level was calculated by normalizing the gene expression levels of tested genes to that of the control 18 S rRNA gene.

CK2α: Fwd: 5’ TAGGGGGTTGGTATCTCGTG3’; Rev: 5’ TGATGTAAGCGACCAGCAAG3’

BMI1: Fwd::5’ CCTTCATTGTCTTTTCCGCCC; Rev:5’ AAGTACCCTCCACAAAGCAC 3’

18 S rRNA: Fwd:5’ TCGATGGGCGGCGGAAAATA; Rev: 5’ TTGGTGAGGTCAATGTCTGCT3’

### Clonal growth assay

48 h post transfection 200 single cells of CP20 or OV90 cells in RPMI medium containing 10% FBS were seeded in the same medium in each well of 6-well plate. After 10 days (CP20) or 14 days (OV90), the colonies were stained with the crystal violet solution (0.75% crystal violet, 50% ethanol, 0.25% NaCl, 1.57% formaldehyde), imaged using Leica EZ4HD (Buffalo Grove, IL 60089 USA). 9 images from 3 independent experiments were quantified using Image J (image processing and analysis in Java, NIH).

### Data analysis and statistics

All the experiments were repeated independently at least 3 times and in triplicate where applicable. Data are expressed as mean ± standard deviation (SD). Comparisons between two groups were evaluated using Student’s *t*-test with equal/unequal variances. For comparisons among multiple groups, we performed ANOVA. If the overall test was significant, we compared each treatment to control with Dunnett's method for multiple comparisons. For densitometry analysis of immunoblots, Image J software (NIH) was used. BMI1 expression levels were compared between normal and primary tumor samples using Wilcoxon rank-sum test. The CK2α detection rates were compared between the groups (normal vs. primary tumor) using Fisher’s exact test. Spearman correlation coefficient was used to assess the correlation between CK2α and BMI1 expression levels among primary tumor samples. *P* < 0.05 was considered statistically significant. All tests were two-sided.

## Results

### Identification of Casein Kinase 2α, a candidate BMI1 kinase

**Fig. 1:**
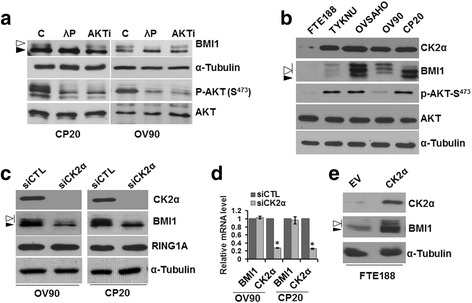
Expression and regulation of BMI1 and CK2α in ovarian cancer cells. **a** Slow migrating phospho-BMI1 bands were reduced upon treatment with λ-Phosphatase but not with AKT inhibitor. OV90 and CP20 cells were treated with 5 μM AKT inhibitor (AKTi) or vehicle (C) for 15 h. Protein extracted from the vehicle treated cells (C) were further subjected to λ-phosphatase (λ-P) treatment or left untreated. Expression of BMI1 and Phospho-AKT (Serine 473) were determined in the C, λ-P and AKTi treated cells. α-Tubulin and AKT were used as a loading control. Slow migrating BMI1 bands that were removed on phosphatase treatment is considered as the phospho species of BMI1 and is indicated by the open arrow head whereas the basal BMI1 is indicated by the closed arrow head. **b** Expression of BMI1, CK2α, and AKT (S473) in a panel of ovarian cancer cells. Protein expression was determined in 4 cell lines representing high grade serous ovarian cancer (TYKNU, OVSAHO and OV90) or cisplatin resistant CP20 and compared to the normal Fallopian Tube Epithelial Cells (FTE188). α-Tubulin and AKT served as a loading. Open and closed arrow heads represent phospho species of BMI1 and basal BMI1 respectively. **c**-**d** Effect of CK2α knockdown on BMI1. **c** OV90 and CP20 cells were either transfected with scrambled siRNA (siCTL) or siRNA against the CK2α gene (siCK2α) and expression of BMI1, RING1A and CK2α were determined by immunoblotting. **d** Relative mRNA expression of BMI1 and CK2α were determined by RT-qPCR. Gene expression of 18 s rRNA was used as an endogenous control. Values represent mean fold change (± standard deviation) over control (siCTL). Open and closed arrow heads represent phospho species of BMI1 and basal BMI1 respectively. **e** Effect of CK2α overexpression on BMI1. FTE188 cells were transfected with CK2α plasmid and 24 h post transfection the expression of BMI1 and CK2α were determined by immunoblotting. Open and closed arrow heads represent phospho species of BMI1 and basal BMI1 respectively

AKT has been identified as the predominant kinase phosphorylating BMI1 on Ser 251, 253, 255 and 316 respectively, resulting in its altered cellular activity [3, 12]. However, inhibition of AKT by specific inhibitors (AKTi) reduced but did not eliminate the characteristic phospho-bands of BMI1 in ovarian cancer cells, CP20 and OV90 (Fig. 1a). That the slower migrating species represented phosphorylated form of BMI1 (Fig. 1a) was confirmed by λ-phosphatase treatment [15]. This suggests that in ovarian cancer cells additional kinases might be involved in phosphorylating BMI1. Stringent bioinformatics analysis (NetPhos2.0 Server [28], cutoff <0.9) of the BMI1 protein sequence revealed nine potential sites of phosphorylation (Table 1) including Ser 253, 255 and 316 that were previously described [3, 12]. Interestingly, all of the previously reported phosphorylation sites of BMI1 lie within the PS/PEST domain (236–326 amino acids), deletion of which increases half-life of the protein [15]. Interrogation of these phosphorylation sites with KinasePhos2 [29] analysis (Table 1) predicted previously reported kinases such as AKT [3, 12], as well as novel candidate kinases such as Ataxia Telangiectasia Mutated (ATM) and Casein Kinase II (CK2) (Table 1). While ATM/ATR dependent recruitment of BMI1 to sites of DNA damage has been reported [4], any association with CK2 remains unknown.

**Table 1:** Bioinformatics analysis of the potential phospho sites on BMI1 protein sequence

Score^a^	Sequence^a^	Site^b^	Predicted kinase
0.994	KFLRSKMDI	S181	PKB/ATM/Aurora/MAPK
0.993	LESDSGSDK	S253	ATM/PLK1/CK2
0.991	PRKSSVNGS	S316	PKB/ATM/Aurora/IKK/CAMK/PLK1
0.987	SDSGSDKAN	S255	ATM/Aurora/CDK
0.987	RPRKSSVNG	S315	ATM/Aurora/IKK/CAMK
0.984	DKANSPAGG	S260	ATM/Aurora/PLK1/MAPK/CK1
0.955	TPVQSPHPQ	S281	ATM/Aurora/MAPK/CDK/CK1
0.951	AANGSNEDR	S110	ATM/CK2
0.918	YLETSKYCP	S50	ATM

The data derived from NetPhos2.0 server and KinasePhos 2 are indicated by ^a^ and ^b^ respectively

CK2 is a serine-threonine kinase comprising of two catalytic (α, α') and two regulatory (β) subunits. CK2 is upregulated in major lethal malignancies where its expression correlates with poor overall survival [16, 18, 19]. Interestingly of these subunits, high levels of CK2α correlated with poor overall survival in ovarian cancer [19]. Using immunoblotting, we determined that CK2α levels were significantly elevated in ovarian cancer cell lines compared to the non-malignant Fallopian Tube Epithelial (FTE188) cells (Fig. 1b). Since BMI1 is reportedly phosphorylated by AKT, we determined the expression of active AKT (Phospho AKT-S^473^) in these cell lines. With the exception of OV90, AKT was significantly phosphorylated in all the cancer cell lines compared to FTE188 (Fig. 1b). Interestingly, the phosphorylated species of BMI1 was present in all the cancer cell lines including OV90 that expressed minimal phospho-AKT, thereby implicating other kinases in phosphorylation of BMI1. Since the expression of CK2α correlated with the phospho-species of BMI1, we next determined if altering cellular levels of CK2α affected phosphorylation of BMI1. Silencing CK2α by siRNA significantly reduced both phospho-species as well as basal BMI1 levels in CP20 and OV90 cells (Fig. 1c). However the PRC1 complex partner of BMI1, RING1A levels remained unchanged (Fig. 1c). Subsequently we confirmed that in CK2α silenced cells, reduction in basal BMI1 level was not due to decreased transcription thus implicating post-translational events (Fig. 1d). These observations were further complemented by expressing CK2α in FTE188 cells that resulted in enhanced basal expression as well as phosphorylation of BMI1 (Fig. 1e). Together these results suggest that CK2α might potentially phosphorylate BMI1 and directly or indirectly regulate its post translational expression.

### CK2α phosphorylates BMI1

**Fig. 2:**
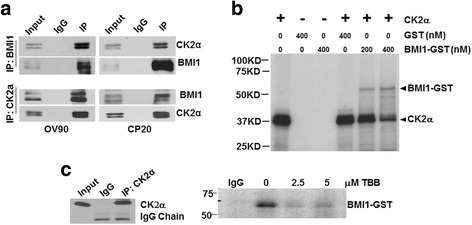
BMI1 is a substrate of CK2α. **a** Co-precipitation of BMI1 with CK2α. Reciprocal immunoprecipitation (IP) assay was performed with BMI1 and CK2α antibodies crosslinked with the agarose resin and immunoprobed with CK2α and BMI1 antibodies respectively. **b** In vitro kinase assay with CK2α and BMI1. In vitro kinase assay was performed with 400nM CK2α, 200nM or 400nM BMI1-GST and radioactive ATP and representative autoradiograph image is presented.. Reaction mixture without substrate (lane 1), only GST protein (lane2), without enzyme (lane 3), and enzyme with GST only (lane 4) served as negative controls. **c** Kinase assay with immunoprecipitated (IP) endogenous CK2α and purified BMI1. CK2α was immunoprecipitated from CP20 cells using agarose A/G beads, the beads was washed and incubated in a kinase assay buffer supplemented with purified BMI1-GST and radiolabeled ATP, in presence or absence of the specific CK2 inhibitor TBB. A representative autoradiograph is provided in the right panel. Efficient Immunoprecipitation is demonstrated in the left panel by immunoblotting a small fraction of the IPed beads with CK2α antibody

To determine if BMI1 is a direct substrate of CK2α, we performed reciprocal co-immunoprecipitation of endogenous BMI1 and CK2α using covalently cross-linked antibodies to protein A/G agarose beads. BMI1 and CK2α co-precipitated from lysates of both CP20 and OV90 cells (Fig. 2a), indicating that BMI1 might be a novel phosphorylation substrate of CK2α.

To confirm these observations, we performed an in vitro kinase assay using purified CK2α, GST-tagged BMI1 and [γ − ^32^P] ATP. The autoradiograph clearly demonstrated that BMI1 was phosphorylated by CK2α (Fig. 2b). Additionally, as previously reported [30], autophosphosphorylation of CK2α was also observed (Fig. 2b). To further confirm these observations we first immunoprecipitated CK2α from cellular lysates of CP20 cells using antibody-conjugated agarose beads (Fig. 2c-left panel). Equal amount of these agarose beads were incubated with recombinant BMI1 and [γ − ^32^P] ATP either in presence or absence of different concentrations of the CK2 inhibitor, TBB (4,5,6,7-tetrabromobenzotriazole) [31]. The autoradiograph clearly demonstrated that BMI1 was phosphorylated by CK2α and the signal significantly diminished with TBB (Fig. 2c-right panel). Together these results demonstrate that CK2α phosphorylates BMI1.

### CK2α phosphorylates BMI1 at S110

**Fig. 3:**
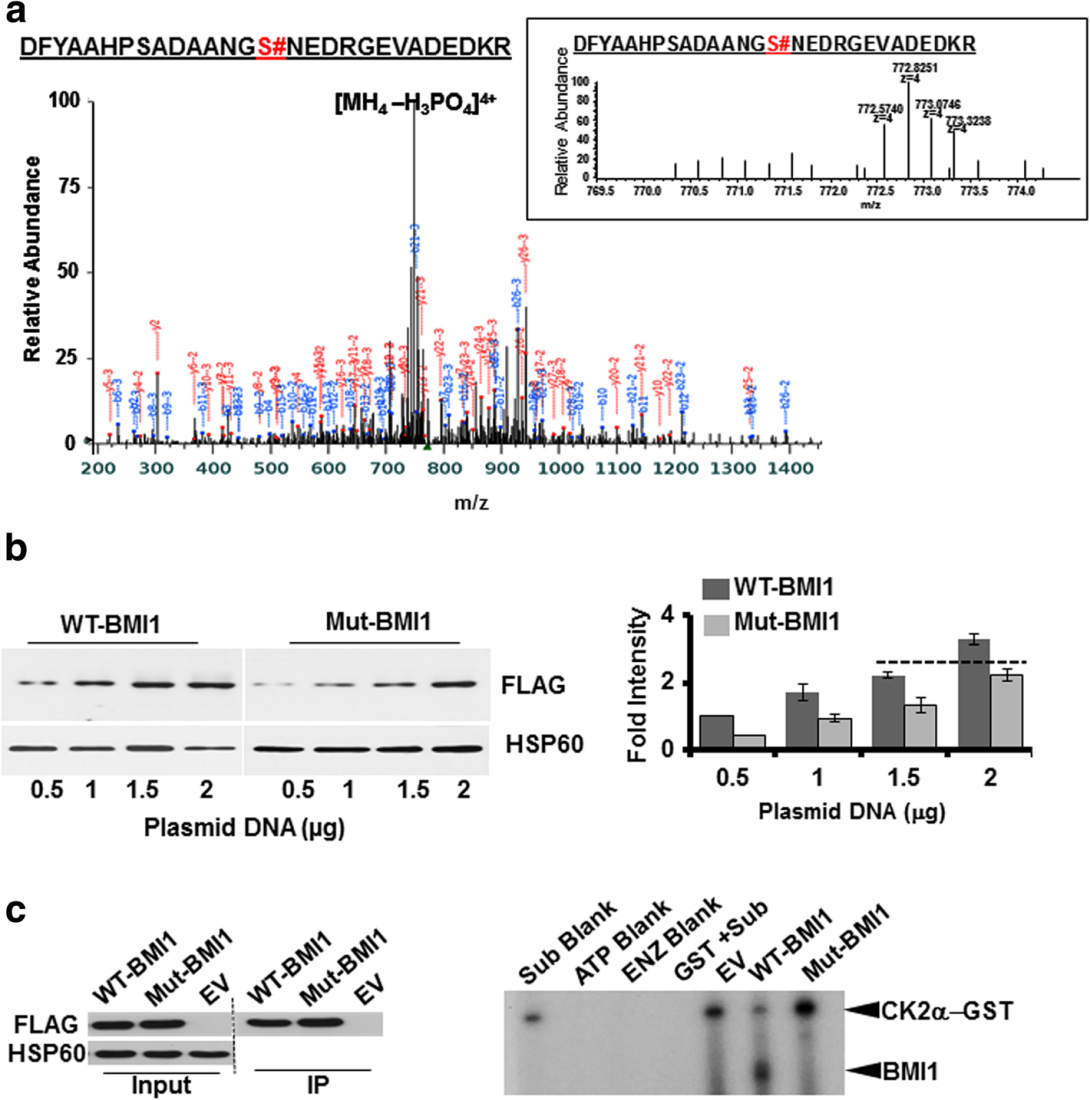
Identification of amino acid residue on BMI1, phosphorylated by CK2α: **a** Kinase assay and Mass-spectrometric analysis. Purified BMI1 and CK2α were allowed to react in a kinase assay buffer supplemented with nonradioactive ATP. The reaction was terminated after 30 min and the sample was sent for mass spectrometric analysis. The spectrum of the BMI1 phospeptide is presented with the extracted ion chromatogram for the peptide with phosphate shown in the inset (indicated by #). **b** Relative expression of WT and mutant BMI1: Phospho mutant BMI1 with substitution of Serine at 110 with Alanine (S110A mutant BMI1) was generated using the wild-type BMI1 (WT-BMI1) as a template for site directed mutagenesis. CP20 cells were transfected with increasing DNA concentration (0.5 μg -2 μg) of wild-type or mutant BMI1 and 30 h post transfection samples were harvested for immunoblotting with FLAG antibody. HSP60 is used as a loading control. Right panel shows a representative immunoblot for the expression of the WT and Mut-BMI1. To determine DNA concentration required to achieve comparable expression between WT and Mut-BMI1, a densitometry analysis of the bands was performed using Image J software (NIH, Bethesda, MD), and the graph was generated by plotting fold intensity vs DNA concentration. The broken line indicates equivalent expression level of WT and Mut-BMI1. **c** Kinase assay with Mutant BMI. CP20 cells were transfected with EV (2 μg), WT (1.5 μg) and Mut-BMI1 (2 μg) and 30 h post transfections cells were harvested for immunoprecipitation with FLAG antibody and agarose beads. A fraction of the agarose beads were processed for immunoblotting to ensure efficient immunoprecipitation and depicted as an immunograph in the left panel. The right panel shows radiograph for kinase assay reaction with the immunoprecipitated beads and in presence/absence of Purified GST-CK2α and radiolabeled ATP

To determine the potential site/s on BMI1 that are phosphorylated by CK2α, full-length purified BMI1 was subjected to the in vitro kinase reaction in presence or absence of purified CK2α and non-radioactive ATP and then analyzed by mass spectrometry (MS). 19% of the detected BMI1 peptides were phosphorylated at Ser110 compared to none without CK2α as shown in the phosphopeptide spectrum (Fig. 3a and Additional file 1: Figure S1). The extracted ion chromatogram for the peptide is shown in the inset of Fig. 3a. By site-directed mutagenesis of the FLAG tagged wild-type BMI1 (WT) construct, we generated a S110A mutant and expressed these constructs in CP20 cells. Immunoblotting and densitometric analysis demonstrated that comparable expression could be achieved by transfection 1.5 μg of WT and 2 μg of the S110A-BMI1 mutant (Mut-BMI1) constructs respectively (Fig. 3b). Next, WT or Mut-BMI1 was immunoprecipitated using anti-FLAG antibody conjugated to protein A/G agarose beads (Fig. 3c, left panel). Equal amount of beads were incubated with full-length GST-tagged CK2α and [γ − ^32^P] ATP for the in vitro kinase assay. Autoradiograph signals were obtained with WT-BMI1 but not with Empty vector (EV) control or with the Mut-BMI1 (Fig. 3c, right panel). Together these results indicate that BMI1 is phosphorylated by CK2α at the S110 residue.

### Mutation at S110 residue decreases half-life of BMI1

**Fig. 4:**
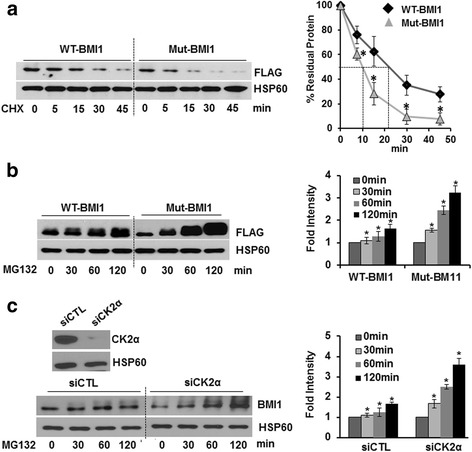
Phospho-mutant BMI1 is intrinsically unstable. **a** Determining the half-life of Wild-type (WT) and S110A mutant BMI1 (Mut-BMI1). The half-life of wild-type and mutant BMI1 in CP20 cells expressing the respective protein was determined by blocking synthesis of new proteins using 100 μg/ml CHX for different time points (0–45 min) and the rate of BMI1 degradation was determined by western blot analysis of FLAG(BMI1) with HSP60 serving as a loading control. Residual BMI1 protein after each time point was normalized to HSP60 using densitometry measurements. The time-dependent decrease of BMI1 was quantified to determine the half-life and is graphically represented in the right panel. The densitometry analysis was performed using Image J software (NIH, Bethesda, MD), and the graph was generated by plotting % residual protein vs time of CHX treatment. **b** Effect of proteosomal inhibition on Wild-type (WT) and S110A mutant BMI1 (Mut-BMI1). 30 h post transfection, CP20 cells were treated with 10 μM MG132, for different time points (0–120 min) to determine the accumulation of WT or mutant BMI1. Left panel represents the immunoblots for FLAG and HSP60 and quantification of the accumulated proteins by densitometry analysis of signal present in respective lanes (normalized to the individual HSP60) is graphically represented in the right panel. **c** Effect of proteosomal inhibition on BMI1 protein accumulation in CK2α silenced cells. CP20 cells were transfected with scrambled siRNA (siCTL) or siRNA against CK2α (siCK2α) and 48 h post transfection, cells were treated with 10 μM MG132, for different time points (0–120 min) to determine the effect of silencing CK2α on MG132 induced accumulation of endogenous BMI1. siCTL or siCK2α transfected cells were harvested after each time point and analyzed for accumulation of the endogenous BMI1 protein by western blot analysis and using HSP60 as a loading control (bottom left panel). Efficient silencing by CK2α siRNA was also determined (top left panel). The accumulated proteins were quantified by densitometry analysis of signal present in respective lanes and by normalizing it to the individual HSP60 and graphically represented in the right panel

Modulation of cellular CK2α levels impacted not only BMI1 phosphorylation but also total protein levels (Fig. 1c-d). In addition, though the plasmid backbones were identical we consistently observed lower expression of S110A compared to wild-type BMI1 (Fig. 3b) suggesting issues with stability of the mutant (non-phosphorylatable) protein. To investigate this possibility, the half-life of the FLAG tagged WT and S110A-BMI1 was determined in CP20 cells using 100 μg/ml cyclohexamide (CHX). As evidenced by immunoblotting and densitometry, WT-BMI1 had a half-life of ~23 min while S110A with a half-life of ~11 min degraded faster (Fig. 4a). Prior studies have reported a half-life of ~25–30 min for WT-BMI1 that is specifically degraded by the 26S proteasome-mediated pathway [15]. To determine if decreased stability of mutant BMI1 was due to proteasome-mediated degradation, we treated plasmid transfected CP20 cells with the proteasome inhibitor MG132 (10 μM) for the indicated times. Compared to the wild-type protein, consistently more S110A-BMI1 accumulated after treatment with MG132 suggesting that the mutation and thereby lack of phosphorylation rendered the protein less stable and more amenable to degradation by the proteasome (Fig. 4b). Similarly, compared to the control more accumulation of endogenous BMI1 could be observed in the MG132 treated CK2α silenced cells (Fig. 4c) further supporting that phosphorylation at S110 affects stability of BMI1.

### Importance of the CK2α/BMI1 axis in ovarian cancer

**Fig. 5:**
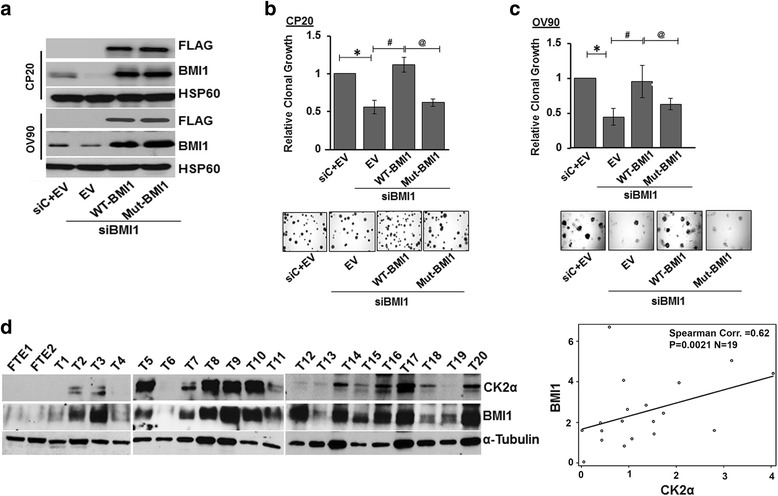
Physiological and clinical relevance of CK2α mediated BMI1 phosphophorylation: **a** Re-expression of exogenous BMI1 in a BMI1 silenced cells. CP20 and OV90 cells are co transfected with FLAG tagged plasmids for WT (wild-type) -BMI1or Mut (S110A) -BMI1 or EV (empty vector) along with siBMI1 (siRNA against the 3’UTR of the BMI1) or siC (scrambled siRNA). Expression of the exogenous and the endogenous BMI1 were probed by immunoblotting with the FLAG and BMI 1 antibodies respectively. HSP60 is used to indicate equal protein loading. **b**, **c** Clonal growth. CP20 and OV90 cells were co-transfected with plasmid and siRNA as described in (A) and 48 h post transfection, cells were recounted and plated as single cells in 35 mm dishes. After 10 (CP20) or 14 (OV90) days, colonies were stained with crystal violet, imaged and 9 images from 3 independent experiments were quantified using ImageJ in a blinded fashion. Top panel depicts a graphical representation of data presented as Relative clonal growth against control (siC + EV) which is considered as 1. Lower panel shows representative images of the colonies. *P* < 0.05 is considered statistically significant and represented with * (SiC + EV vs EV + siBMI1); # (EV + siBMI1 vs WT-BMI1 + siBMI1); @ (WT-BMI1 + siBMI1 vs Mut-BMI1 + siBMI1). **d** Coexpression of CK2α and BMI1 in primary tissues. Protein was extracted from the 20 primary tumors (T1 to T20) and 2 normal fallopian tube epithelial (FTE 1, 2) samples as described in the material and methods section and were quantified by densitometry analysis using Image J. Expression of CK2α and BMI1 were individually normalized to their respective α-tubulin levels. Left Panel represents immunograph for BMI1 and CK2α expression in primary tissues. Right panel represents scatterplot of CK2α vs. BMI1 with overlaid linear regression line among tumor tissue samples (*N* = 19). Spearman correlation coefficient is 0.62 (*p* = 0.0021)

BMI1 confers clonal self-renewal property to cells. Therefore using clonal growth assays, we determined the effect of WT or S110A-BMI1 in OV90 and CP20 cells. Simultaneously, endogenous BMI1 was silenced by a 3'UTR targeted siRNA and comparable levels of WT or S110A-BMI1 was expressed in these cells. Significant knockdown of endogenous BMI1 by siRNA and re-expression of the FLAG tagged WT and S110A-BMI1 was confirmed by immunoblotting (Fig. 5a). Compared to the control, significant decrease in clonal growth was observed in the BMI1 silenced cells (~55% in OV90 and ~45% in CP20; Fig. 5b-c). Re-expression of WT-BMI1 almost completely rescued clonal growth in endogenous BMI1 silenced cells while the S110A-BMI1 had no effect (Fig. 5b-c).

Based on these results we posited that if CK2α mediated BMI1 phosphorylation led to protein stability, then a correlation between CK2α and BMI1 protein levels would be expected in ovarian cancer clinical specimens. Therefore, we analyzed BMI1 and CK2α levels in a panel of twenty high-grade serous ovarian tumors and two normal fallopian tube epithelial (FTE) tissues [32] by immunoblotting. BMI1 expression was significantly higher in primary tissues (*p* = 0.0398) and CK2α was detected in a significantly higher proportion of primary tissues (*p* = 0.013). Remarkably within the tumor samples, significant correlation between CK2α and BM11 expression was observed (Fig. 5d, Spearman correlation coefficient is 0.62, *p* = 0.0021). While patients with higher expression of both BMI1 and CK2α showed a trend towards shorter progression free survival, the data did not reach statistical significance likely due to the limited sample size (Additional file 1: Figure S2). Together, these results demonstrate that phosphorylation of BMI1 at Ser110 by CK2α is important for both stability and functionality of the protein.

## Discussion

Given the important role of BMI1 in cancer biology it is important to investigate its post-translational modifications because they confer functional diversity to proteins and few such modifications of BMI1 have been previously described. Here we report that CK2α by phosphorylating at the Ser110 residue regulates stability of the BMI1 protein.

Interestingly, all of the previously reported phosphorylation sites of BMI1 lie within the C-terminal PS/PEST domain (236–326 amino acids) and involve AKT [3, 12]. Though bioinformatics analysis predicted a number of potential kinases that could phosphorylate BMI1, we focused on CK2α because the hetero-tetrameric CK2 complex with constitutive kinase activity is predominantly present in the nucleus like BMI1. Also, ovarian cancer cells that expressed CK2α but minimally activated AKT maintained phosphorylated species of BMI1. Following prediction, we showed that CK2α reciprocally co-precipitated with BMI1. Using in vitro kinase assays and Mass spectrometry we confirmed that CK2α phosphorylates BMI1 at the Ser110 residue.

Prior reports indicate that protein turnover of BMI1 is regulated by βTrCP recognizing a degron motif within the PS/PEST domain, where all the previously described phosphorylation sites reside [33]. Deletion of the PS/PEST domain thus increases the half-life and stability of the protein [15]. Other mechanisms of BMI1 stability include direct association with the FAL1 long noncoding RNA resulting in increased clonogenicity and tumorigenicity in an ovarian cancer model [34]. Phosphorylation can differentially regulate proteosomal degradation [35–38]. Our results suggest that phosphorylation at Ser110 by CK2α stabilizes BMI1 because mutation to non-phosphorylatable alanine significantly reduces half-life of the protein resulting in increased accumulation upon addition of MG132 indicating proteasomal degradation. Phosphorylation-mediated alteration in protein conformation resulting in reduced ubiquitination or enhanced interaction with other stabilizing proteins, akin to many other cellular proteins [39, 40] may be envisioned.

## Conclusions

Our results, generally, demonstrate a correlation between expression of CK2α and phosphorylated BMI1 in ovarian cancer cell lines and patient tissues. Interestingly previous reports suggest that expression of both CK2α and BMI1 is elevated in ovarian cancer and correlates with poor overall survival (19, 20). Also, in vivo studies in mice bearing A2780-xenografts, confirmed that antitumor efficacy could be enhanced by combining the CK2 inhibitor with cisplatin, carboplatin, or gemcitabine [41]. However, in these studies, the expression of BMI1 was not determined. We find that re-expression of phosphorylatable but not non-phosphorylatable BMI1 rescues clonal growth in endogenous BMI1 silenced ovarian cancer cells leading us to speculate that CK2α-mediated phosphorylation stabilizes BMI1 and promotes its oncogenic function.

## Additional file

Additional file 1: Figure S1. The spectrum obtained from MS analysis of Phospheptide of BMI1 (DFYAAHPSADAANGS#NEDRGEVADEDKR). **Figure S2.** Association between BMI1+ CK2α (categorized into “high BMI1 + high CK2α” vs “others”) and patient survival (PFS). Expression of CK2α and BMI1 in the ovarian cancer patient samples (*N* = 20) were determined by immunoblotting, quantified by densitometry analysis as described in the “methods section” and grouped as high BMI1/high CK2α expressers versus all others. While PFS was worse in the high BMI1 + high CK2α group, the result was not statistically significant (*P* = 0.4), possible due to small sample size. (DOCX 78 kb)

## Abbreviations

BMI1: B lymphoma Mo-MLV insertion region 1 homolog
CK2α: Casein kinase 2 alpha subunit
FFPE: Formalin Fixed Paraffin Embedded
FTE: Fallopian Tube Epithelial
PRC1: Polycomb repressor complex 1
RT-PCR: Reverse Transcription and Polymerase Chain reaction
