# Supplementary material for: BMI1, a new target of CK2α
Source: Mol Cancer. 2017 Mar 7;16:56. doi: 10.1186/s12943-017-0617-8 (PMC5341428; doi:10.1186/s12943-017-0617-8)
Supplement: Additional file 1: Figure S1. — The spectrum obtained from MS analysis of Phospheptide of BMI1 (DFYAAHPSADAANGS#NEDRGEVADEDKR). Figure S2. Association between BMI1+ CK2α (categorized into “high BMI1 + high CK2α” vs “others”) and patient survival (PFS). Expression of CK2α and BMI1 in the ovarian cancer patient samples (N = 20) were determined by immunoblotting, quantified by densitometry analysis as described in the “methods section” and grouped as high BMI1/high CK2α expressers versus all others. While PFS was worse in the high BMI1 + high CK2α group, the result was not statistically significant (P = 0.4), possible due to small sample size. (DOCX 78 kb) [file 12943_2017_617_MOESM1_ESM.docx]

Additional file


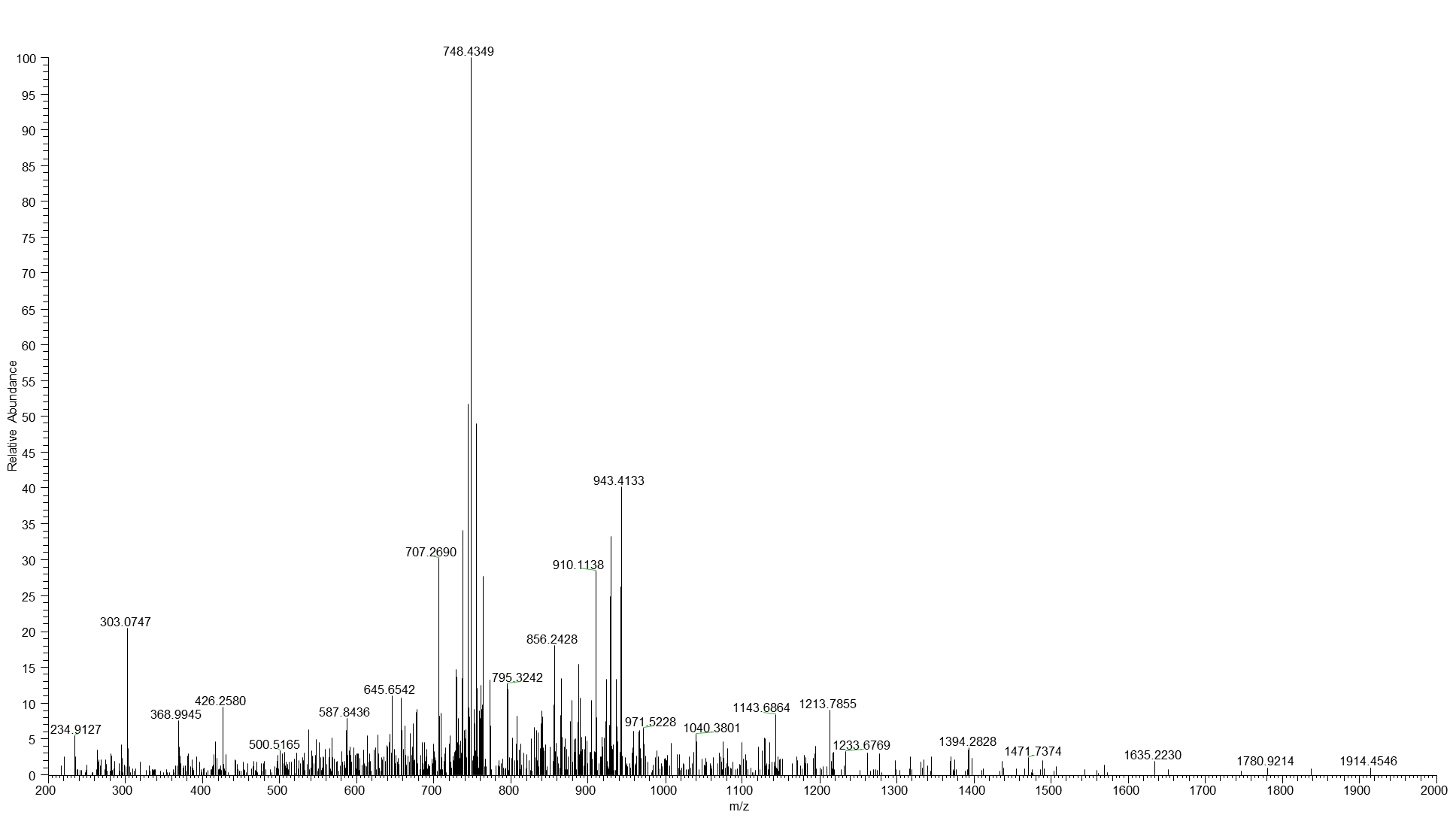


Figure S1: The spectrum obtained from MS analysis of Phospheptide of BMI1 (DFYAAHPSADAANGS#NEDRGEVADEDKR)

Figure S2: **Association between BMI1+ CK2α (categorized into “high BMI1 + high CK2α” vs “others”) and patient survival (PFS).**

Expression of CK2α and BMI1 in the ovarian cancer patient samples (N=20) were determined by immunoblotting, quantified by densitometry analysis as described in the “methods section” and grouped as high BMI1/high CK2α expressers versus all others. While PFS was worse in the high BMI1 + high CK2α group, the result was not statistically significant (P=0.4), possible due to small sample size.
